# Sensorimotor measures predict longitudinal cognitive decline and correlate with CSF and plasma biomarkers

**DOI:** 10.1093/geroni/igaf122.4189

**Published:** 2025-12-31

**Authors:** Semere Bekena, Ramkrishna Kumar Singh, Yiqi Zhu, David Carr, Ganesh Babulal

**Affiliations:** Washington University School of Medicine in St. Louis, St louis, Missouri, United States; Washington University School of Medicine in St. Louis, St. Louis, Missouri, United States; Washington University School of Medicine in St. Louis, Washington Universury, Missouri, United States; Washington University in St. Louis, St Louis, Missouri, United States; Washington University School of Medicine in St. Louis, SAINT LOUIS, Missouri, United States

## Abstract

Identifying early markers of cognitive decline in cognitively normal older adults is critical for dementia prevention. Sensorimotor measures such as gait speed, grip strength, and reaction time may serve as accessible, sensitive indicators of current and future cognitive impairment. This study examined associations between baseline sensorimotor function and cognitive performance and decline, assessed by the Preclinical Alzheimer Cognitive Composite (PACC), in cognitively normal older adults. In this prospective cohort study, 246 cognitively normal older adults from the DRIVES Project completed baseline assessments of grip strength, gait speed, simple reaction time, and PACC scores. Linear mixed-effects models adjusted for demographic, genetic, and neighborhood depravity level. Cross-sectional analyses examined associations between sensorimotor measures and cerebrospinal fluid (CSF) biomarkers of amyloid and tau pathology, and plasma neurofilament light chain (NfL), where available. Participants (mean age: 74.9 ± 5.17 years; 48.8% female) followed on average for 4 years had a mean baseline PACC score of 1.06 ± 0.50. Cross-sectionally, slower gait speed was associated with higher CSF tau/Aβ42 ratio (p = 0.027), CSF tTau/Aβ42 ratio (p = 0.009), and plasma NfL (p = 0.024). Slower reaction time predicted lower baseline PACC scores (p = 0.028). Longitudinally, low grip strength (p = 0.002) and slow gait speed (p < 0.001) predicted lower cognitive performance, with slow gait speed predicting faster decline (p = 0.015). In conclusion, sensory-motor function measures are associated with current and future cognitive performance, supporting their role in early identification of older adults (65 and above) at risk for cognitive decline.

